# Fibroblast growth factors signaling in bone metastasis

**DOI:** 10.1530/ERC-19-0472

**Published:** 2020-05-04

**Authors:** Estefania Labanca, Elba S Vazquez, Paul G Corn, Justin M Roberts, Fen Wang, Christopher J Logothetis, Nora M Navone

**Affiliations:** 1 Department of Genitourinary Medical Oncology and the David H. Koch Center for Applied Research of Genitourinary Cancers, The University of Texas MD Anderson Cancer Center, Houston, Texas, USA; 2 Laboratorio de Inflamación y Cáncer, Departamento de Química Biológica, Facultad de Ciencias Exactas y Naturales, Universidad de Buenos Aires, Buenos Aires, Argentina; 3 CONICET – Universidad de Buenos Aires, Instituto de Química Biológica de la Facultad de Ciencias Exactas y Naturales (IQUIBICEN), Buenos Aires, Argentina; 4 Institute of Biosciences and Technology, Texas A&M Health Science Center, Houston, Texas, USA

**Keywords:** prostate cancer, bone metastasis, fibroblast growth factors, fibroblast growth factor receptors

## Abstract

Many solid tumors metastasize to bone, but only prostate cancer has bone as a single, dominant metastatic site. Recently, the FGF axis has been implicated in cancer progression in some tumors and mounting evidence indicate that it mediates prostate cancer bone metastases. The FGF axis has an important role in bone biology and mediates cell-to-cell communication. Therefore, we discuss here basic concepts of bone biology, FGF signaling axis, and FGF axis function in adult bone, to integrate these concepts in our current understanding of the role of FGF axis in bone metastases.

## Introduction

Development of metastases is a complex and demanding process cancer cells must overcome to successfully colonize remote organ sites (Sethi & Kang 2011). Stephen Paget’s 1889 work proposed that metastasis depends on the crosstalk between cancer cells (the ‘seeds’) and specific organ microenvironments (the ‘soil’). This hypothesis has been used to account for the non-random and cancer-specific distribution of metastases. Prostate cancer is one of the most striking examples of the selectivity of cancer cells for specific sites of metastasis. Indeed, more than 80% of advanced prostate cancer will develop bone metastases and the majority will be bone forming (Loberg *et al.* 2005). Also, targeting bone metastases in prostate cancer with a bone homing α-emitting radiopharmaceutical lengthens survival (Parker *et al.* 2013). Thus, these observations suggest that bone metastases play a central role in prostate cancer progression. In contrast, other malignancies have a lower incidence of bone metastases (e.g. breast (50–60%) (Hess *et al.* 2006), renal (35%), lung (35%), liver (13%), and rectal (10%) carcinoma (Hess *et al.* 2006, Freeman *et al.* 2015)) and only prostate cancer has bone as a single, dominant metastatic site (Hess *et al.* 2006). Additionally, multiple myeloma, a B cell malignancy, is the second most common haematological malignancy and, characteristically, involves bone during progression (Panaroni *et al.* 2017).

The contribution of bone metastases to the clinical morbidity of solid tumors has prompted efforts to better understand the mechanism of cancer metastases to bone. As a result, many factors implicated in bone metastases have been identified. Prominent among these areas of study is the fibroblast growth factor (FGF) signaling axis, which has been shown to be central to the metastatic progression in bone of some tumors (e.g. prostate cancer).

The FGF axis has an important role in bone biology. This axis mediates cell-to-cell communication physiologically in several systems. Therefore, the role of FGF axis in cancer metastases needs to be studied with an understanding of its function in bone and cell biology. This will enable a more rational design of therapies. We will, therefore, introduce basic concepts of bone biology and FGF/FGF receptor (FGFR) axis function, followed by a discussion of evidences implicating this pathway in the pathogenesis of bone metastases in different malignancies.

## Bone development and normal bone biology

In the embryo, bone formation involves the conversion of preexisting mesenchyme into bone tissue. Briefly, skeletogenesis starts with mesenchymal condensation in all prospective bones. The bone tissue is then formed by two different mechanisms: endochondral (axial and appendicular bones) and intramembranous ossification (flat bones of the face, most of the cranial bones, and the clavicles). During endochondral ossification, condensation leads to the formation of a complete cartilaginous skeleton that will eventually be replaced by bone (Rodan 2003). In intramembranous ossification, mesenchymal condensation is followed directly by ossification centers. Cells then assume osteoblastic features and start depositing bone matrix that will go on to mineralize and form the bones. Osteoblasts embedded in the bone matrix become osteocytes (Rodan 2003, Dallas *et al.* 2013). The commitment of mesenchymal stem cells and differentiation into osteoblasts requires Runt-related transcription factor 2 (RUNX2) and osterix, master transcription factors that regulate several genes, such as type I collagen, bone sialoprotein, osteopontin (OPN), transforming growth factor beta (TGFβ), and osteocalcin. The regulation of bone formation involves several factors, including TGFβs, bone morphogenetic proteins (BMPs), FGFs, and Wnt signaling, all of which were shown to regulate cell differentiation and survival in a spatiotemporal manner (Berendsen & Olsen 2015, Ornitz & Marie 2015). In summary, a network of signaling molecules governs bone morphogenesis. Among them, FGF and their receptors were identified as relevant players in bone formation, and some functional redundancies and complementary roles between different FGFRs throughout osteogenesis have been determined (Karuppaiah *et al.* 2016).

During adulthood, bone undergoes continuous remodeling via resorption and replacement at basic multicellular units (BMUs). This process of bone remodeling is critical for bone homeostasis in response to structural and metabolic demands and is strictly controlled through a complex cell communication network involving signals between cells of the osteoblastic and osteoclastic lineages at each BMU (Sims & Martin 2014). In this process, the multifunctional osteocytes regulate osteoblasts and osteoclasts function, therefore, having key roles in bone homeostasis (Dallas *et al.* 2013). Many factors mediating stimulatory and inhibitory signals contribute to coupling the processes of bone formation and resorption, including oncostatin M, parathyroid hormone-related protein (PTHrP), sclerostin, matrix-derived TGFβ, insulin growth factor 1 (IGF-1), cardiotrophin-1, semaphorin 4D/3B, sphingosine 1-phosphate, ephrinB2 and ephrinB4, receptor activator of nuclear factor kappa-B ligand (RANKL), WNT5a, osteoprotegerin, and T cell-derived interleukins (ILs).

More recently, evidence indicates that bone-forming mature osteoblast and bone-resorptive mature osteoclast functions are also regulated via direct cell–cell contact between these cell types (Furuya *et al.* 2018).

These pathways and *bona fide* cell-to-cell interactions in bone are hijacked by cancer cells during the metastatic process. Depending on the specific interaction that occurs between cancer cells and bone cells, bone metastases can be osteoblastic (e.g. prostate cancer) or osteolytic (e.g. multiple myeloma). However, in the majority of bone metastases both components (osteolytic and osteoblastic) are present at different levels.

## Fibroblast growth factor, fibroblast growth factor receptor family

**Figure 1:**
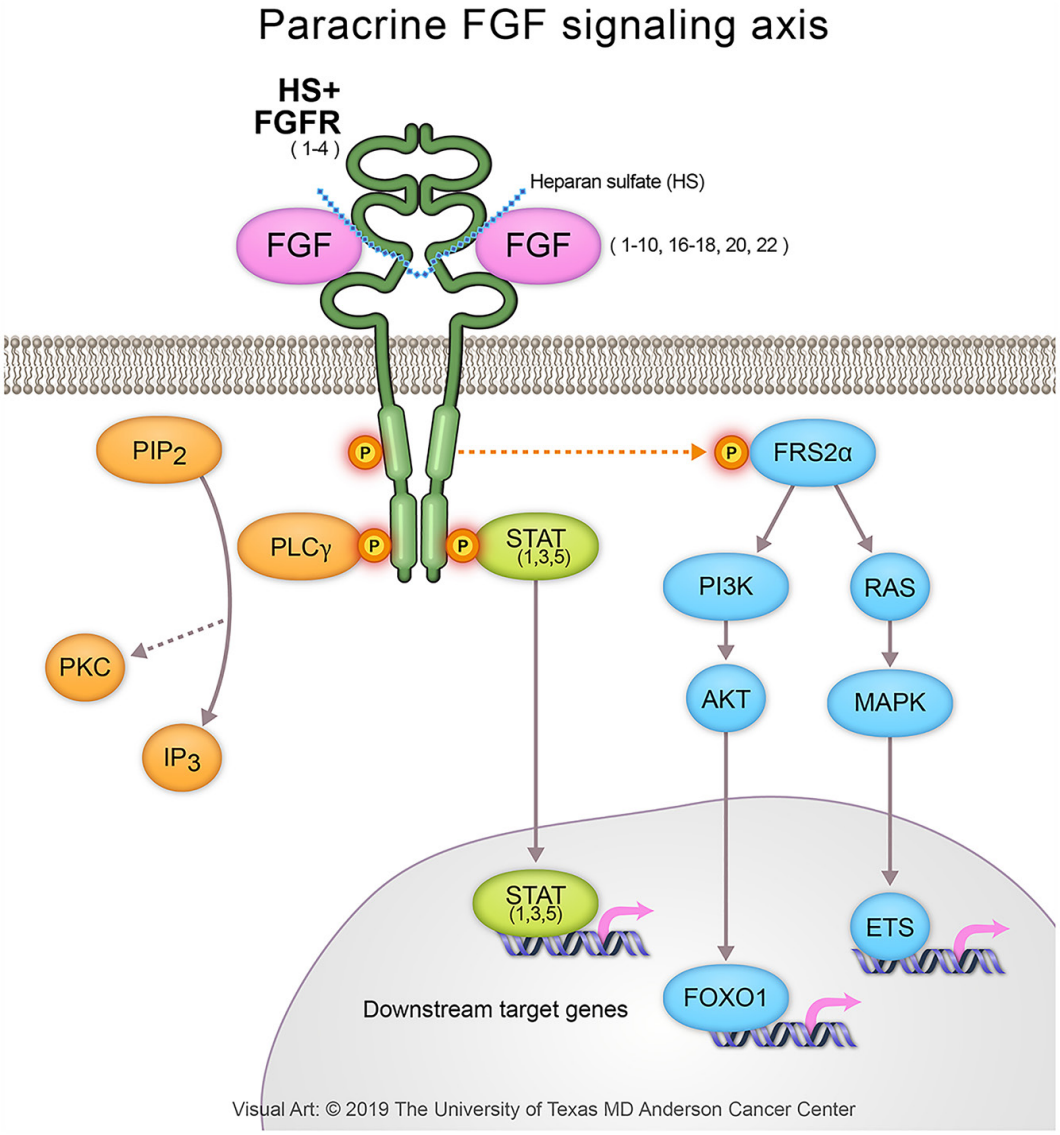
Paracrine FGF signaling pathways. A ternary FGF-FGFR-HS complex results from the binding of canonical FGF to FGFR with HS. This complex then activates the FGFR intracellular tyrosine kinase domain by phosphorylation of specific tyrosine residues. The FRS2α, a major FGFR kinase substrate, is phosphorylated by the activated FGFR kinase. Phosphorylated FRS2α then recruits the RAS/MAPK pathway. MAPK activates members of the ETS transcription factor family and negative regulators of the FGF signaling pathways. Phosphorylated FRS2α also recruits the enzyme PI3K, which then phosphorylates AKT. AKT has multiple activities including the activation of forkhead box protein O1 (FOXO1) transcription factor. Activated FGFR kinase recruits and activates the enzyme PLCγ as well, which produces inositol triphosphate (IP_3_) and phosphatidylinositol bisphosphate (PIP_2_). Also, FGFR kinase activates STAT1, 3, and 5, which mostly regulate gene expression in the nucleus. Copyright held by, and used with permission of, The University of Texas MD Anderson Cancer Center.

The FGF axis is a highly conserved complex signaling pathway, composed of various FGFs, classified as follows: canonical (paracrine), hormone-like (endocrine), and intracellular (intracrine). The canonical subfamily comprises 15 known receptor-binding ligands (FGF1–10,16–18, 20, and 22) (Li *et al.* 2016) that interact with four tyrosine kinase membrane receptors, FGFRs. This interaction in the paracrine signaling requires heparan sulfate (HS), which leads to activation of the FGFR kinases. Current evidence indicates that FGFR kinase activation is followed by phosphorylation of FGFR substrate 2α (FRS2α), recruitment of phospholipase Cγ (PLCγ), and activation of downstream cascades and networks (e.g. mitogen activated protein kinase (MAPK), phosphatidylinositol-3-kinase (PI3K)/protein kinase B (AKT), and signal transducer and activator of transcription (STAT)) (Fig. 1) (Ornitz & Itoh 2015). FGFR signaling can be modulated by different mechanisms including negative regulators (e.g. Sprouty) and receptor internalization and degradation (Ornitz & Itoh 2015).

**Figure 2:**
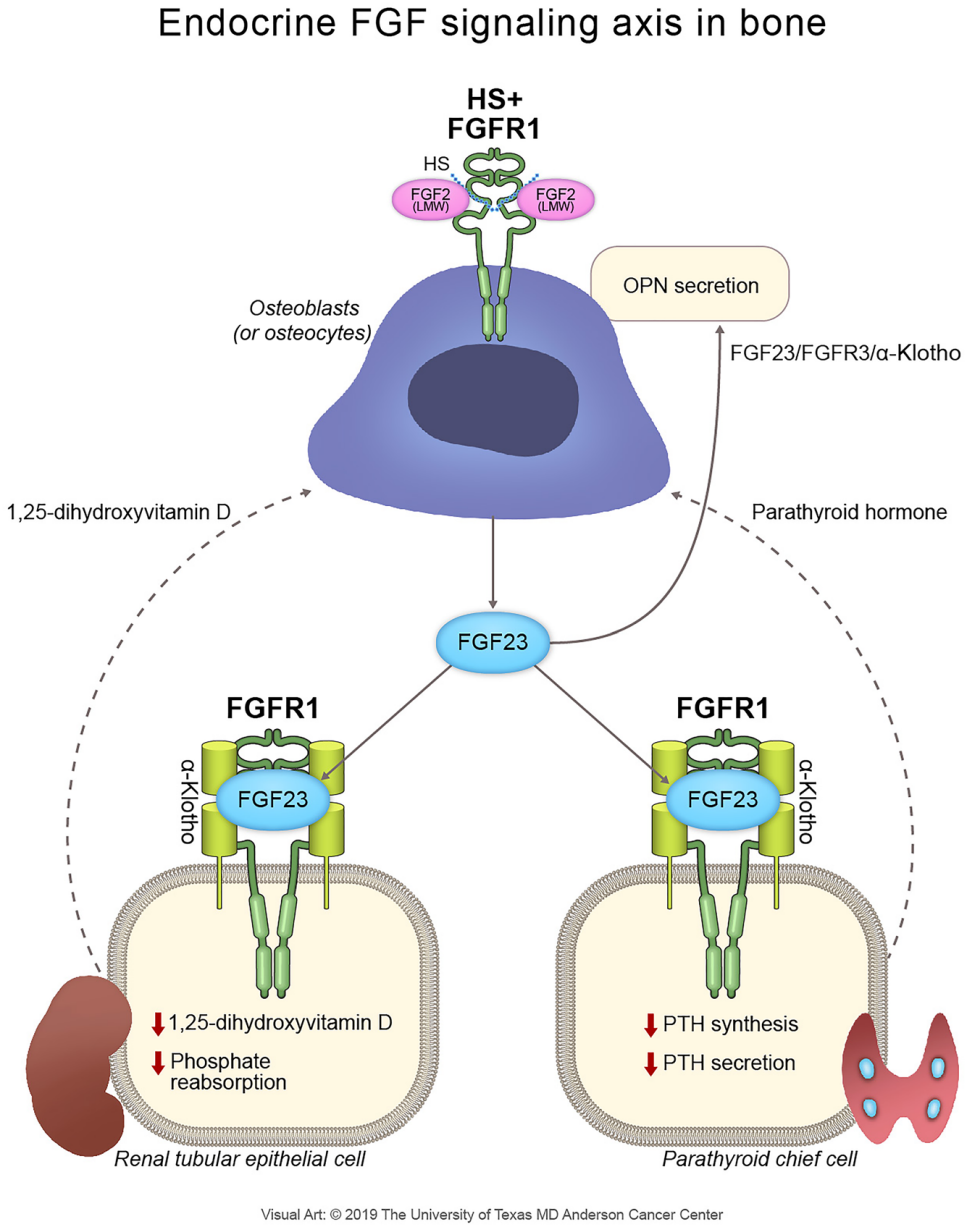
FGF23 endocrine and autocrine/paracrine actions. In osteoblasts/osteocytes, activation of FGFR1 by formation of a ternary complex with LMW-FGF2 and HS in the membrane (or FGF23 binding to intranuclear FGFR1, not shown) induces FGF23 expression. FGF23 in the kidney forms a ternary FGF23-FGFR-Klotho complex, leading to activation of the FGFR tyrosine kinase and inhibition of phosphate reabsorption and reduction of circulating levels of 1,25-dihydroxyvitamin D. 1,25-dihydroxyvitamin D in turn induces FGF23 production by osteoblasts. FGF23 is also thought to inhibit PTH production by the parathyroid gland. Finally, FGF23 regulates OPN secretion in osteoblastic cells, which is a potent regulator of the mineralization process. For brevity, the figure depicts osteoblasts only. Copyright held by, and used with permission of, The University of Texas MD Anderson Cancer Center.

In addition to the paracrine canonical FGFs, there are three FGFs, namely FGF19, 21, and 23, that function as endocrine factors and are believed to require protein cofactor αKlotho, βKlotho, or the Klotho-related protein, for receptor binding and activation due to their lower affinity for HS (Fig. 2) (Ornitz & Itoh 2015). Also, the intracrine FGF subfamily, FGF11–14, encodes intracellular FGFs, which are non-signaling proteins that serve as cofactors for voltage-gated sodium channels and other molecules (Ornitz & Itoh 2015).

The complexity of this axis is further increased by FGFRs alternative splicing, producing isoforms with differential ligand specificity and spatial lineage expression, as well as the complexity of HSs (Li *et al.* 2016). Lastly, an additional member of the family is FGFR-like 1 (FGFRL1) or FGFR5, which lacks the cytoplasmic tyrosine kinase domain (Kahkonen *et al.* 2018).

The FGF pathway plays a central role in various processes that include embryonic and organ development, wound healing, and carcinogenesis (Teven *et al.* 2014). FGF signaling regulates mitogenesis, differentiation, angiogenesis, survival, and motility/invasiveness, among other cellular biological processes, and is integral to normal bone development and function (Ornitz & Marie 2015, Li *et al.* 2016).

## Role of FGF in bone homeostasis

The discovery that FGFR mutations are associated with specific skeletal abnormalities in humans has established the relevance of this pathway in bone development and homeostasis (Ornitz & Marie 2015). However, the effects of FGF/FGFR signaling in osteogenesis are complex, as they depend on which FGFs and FGFRs are expressed, the stage of maturation of the target cells, and the microenvironment (e.g. availability of HS). Genetically engineered mice (GEM) studies shed some light on the role of the FGF axis in bone biology.

Both genes, *Fgfr1* and *Fgfr2*, are expressed in the mouse osteoprogenitor lineage. *Fgfr1* knockout in mature osteoblasts resulted in increased bone mass and osteoblast number in mice (Jacob *et al.* 2006, Zhang *et al.* 2014). *Fgfr1* deletion in osteochondro-progenitors results in increased proliferation and delayed differentiation of osteoblasts (Jacob *et al.* 2006). The collective data suggest that FGFR1 promotes the differentiation of mesenchymal progenitors, while inhibiting the proliferation of mesenchymal progenitors into preosteoblasts. Also, FGFR1 is thought to inhibit the maturation and mineralization of osteoblasts (Jacob *et al.* 2006, Su *et al.* 2014).

Congenital *FGFR2* mutations in humans are associated with craniosynostosis (Karuppaiah *et al.* 2016) and bent bone dysplasia among other skeletal disorders, implicating this receptor in bone development (Neben *et al.* 2017). Conditional knockout studies targeting *Fgfr2* in mice suggest that this receptor is also involved in postnatal bone growth (Karuppaiah *et al.* 2016). However, the mechanism underlying the decreased bone growth observed in the *Fgfr2* gene inactivation studies is not clear.

*Fgfr1* and *Fgfr2* genes have considerable overlap in their expression patterns in mice. To study possible functional redundancies, a double (*Fgfr1* and *Fgfr2*), osteoblast-specific, conditional knockout mouse was generated. These mice appear normal at birth, but show severe postnatal growth defects and impaired longitudinal bone growth, suggesting an important role for FGF signaling in bone formation after birth (Karuppaiah *et al.* 2016). Further, reduction of *Fgfr1/2* expression in osteoblasts resulted in upregulation of *Fgf9*, *Fgf18*, and parathyroid hormone-like peptide (*Pthlh*) genes, which led to increased expression and signaling of *Fgfr3* in growth plate chondrocytes and suppression of chondrocyte proliferation (Karuppaiah *et al.* 2016). Together, these results suggest that, in mice, *Fgfr3* is expressed in proliferating and prehypertrophic chondrocytes and functions to inhibit postnatal chondrogenesis (Ornitz & Marie 2015). The inhibitory activity of FGFR3 on growth plate chondrocytes explains the pathogenic consequences of gain-of-function mutations in *FGFR3* in suppressing pre-pubertal skeletal growth in achondroplasia and related chondrodysplastic disorders. Although much is known about the signals downstream of FGFR3 in chondrocytes, the mechanisms that regulate FGFR3 expression and activation and that coordinate osteogenesis and chondrogenesis are poorly understood (Ornitz & Marie 2015).

Subsequent studies using conditional inactivation of *Fgfr1* and* Fgfr2* in osteoblasts showed that 6- and 12-week-old mice lacking both receptors or only FGFR1 had an increased bone mass phenotype accompanied with impaired material properties. This phenotype was found to be preceded by a remarkable decrease in viable osteocytes and a parallel activation of the Wnt/β-catenin signaling pathway. Similar findings were observed after conditional inactivation of *Fgfr1* and *Fgfr2* in osteocytes. These data suggest that FGFR1 and/or FGFR2 expression in mature osteoblasts/osteocytes is required for osteocyte survival and regulation of bone mass during postnatal bone growth (McKenzie *et al.* 2019). A previous report indicated that conditional knockout of *Fgfr1* in osteocytes in mice results in decreased osteocyte-specific gene expression but no overt skeletal phenotype (Xiao *et al.* 2014). This discrepancy suggests that, in osteocytes, FGFR1 and 2 cooperate for these cells’ survival.

The specific roles of FGF ligands in bone biology post birth are not completely understood. Cell-based and GEM studies implicate FGF2 (one of the most studied FGF ligands) in osteogenesis (Montero *et al.* 2000, Su *et al.* 2014). However, different FGF2 isoforms (low molecular weight (LMW) and high molecular weight (HMW)) seem to have opposite effects on bone mass (Xiao *et al.* 2010, 2018). Briefly, LMW-FGF2 induces bone mass in mice by modulating the Wnt/β-catenin signaling pathway (Xiao *et al.* 2009). In contrast, HMW-FGF2 is a negative regulator of osteoblast differentiation and matrix mineralization (Xiao *et al.* 2013, Homer-Bouthiette *et al.* 2014).

Both, LMW- and HMW-FGF2 induce *Fgf23* promoter activity, but through different mechanisms: by activating cell surface and intranuclear FGFR1, respectively (Han *et al.* 2015). FGF23 is expressed mainly by osteoblasts and osteocytes (Martin *et al.* 2011) and controls phosphate homeostasis and bone mineralization via endocrine actions in its main target organ, the kidney, after formation of the ternary FGF-FGFR-Klotho complex (Quarles 2012, Feng *et al.* 2013). FGF23 inhibits renal phosphate reabsorption. Also by its effect in the kidney, excess FGF23 reduces circulating levels of 1,25-dihydroxyvitamin D (Quarles 2012). There is also evidence suggesting the existence of a parathyroid hormone (PTH)–bone feedback loop in which PTH stimulates FGF23 expression in bone and FGF23 inhibits PTH production by the parathyroid gland (Fig. 2) (Quarles 2012). Finally, FGF23 also locally regulates bone mineralization acting through FGFR3 in a Klotho-independent manner. In this case, FGF23 regulates OPN secretion in osteoblastic cells, which is a potent regulator of the mineralization process (Fig. 2) (Murali *et al.* 2016).

Combined *in vitro* and *in vivo* studies suggest that coordinated FGF and extracellular signal-regulated kinases 1/2 (ERK1/2) signaling regulates the expression of dentin matrix acidic phosphoprotein 1 (*Dmp1*) in osteocytes. DMP1 is abundantly expressed in osteocytes and plays a critical role in osteocyte differentiation and mineralization. Furthermore, DMP1 constitutes an added regulatory mechanism of FGF23 systemic levels and, in turn, of phosphate metabolism (Kyono *et al.* 2012).

Once activated, FGF signaling can be regulated by receptor internalization and degradation. This mechanism involves the interaction of activated FGFR with multiple proteins, including the docking protein FRS2α and the ubiquitin ligase c-CBL, an adaptor protein that mediates FGFR ubiquitination after ligand binding. This mechanism of down-regulation of activated FGFR signaling is prevalent in osteoblasts, highlighting the important role of c-CBL in the control of osteoblastogenesis (Ornitz & Marie 2015).

FGF signaling interacts with other pathways involved in osteogenesis, most notably with BMPs and the Wnt canonical pathway. Briefly, *in vitro* and *in vivo* studies indicate that FGFs enhance canonical BMP2 signaling and induce β-catenin nuclear accumulation in osteoblasts, thus regulating the fate and differentiation of mesenchymal stem cells (Miraoui & Marie 2010, Ornitz & Marie 2015).

Furthermore, FGF2 is necessary for the positive effects of PTH on osteoblast proliferation and differentiation (Ornitz & Marie 2015). In turn, PTH stimulates *Fgf2*, *Fgfr1*, and *Fgfr2* in osteoblasts.

The FGF axis controls bone remodeling by regulating osteoclast activation and function as well. FGF2 induces osteoclast precursor proliferation and stimulates bone resorption through the activation of FGFR1 and MAPK. FGF18 can induce RANKL and cyclooxygenase-2 expression in osteoblasts, which in turn will induce osteoclast formation and function. *In vivo* studies indicate that FGFR1 and FGFR3 contribute to osteoclast activity (Ornitz & Marie 2015). Further, mice with *Fgfr1* inactivation in osteoclast and osteoclast precursors are normal at birth but have abnormal bone remodeling and increased bone mass (Lu *et al.* 2009).

In summary, the FGF axis is a key player in osteogenesis and its function is multifaceted and context dependent, with the effects of particular components, as well as interacting proteins, varying according to the specific microenvironment and stage of bone development. Numerous downstream signaling cascades triggered by the interaction between FGFs and FGFRs in association with other pathways regulate the different steps in osteoblast maturation.

Overall, the previously mentioned studies emphasize the complexity of bone formation dynamics, which require a tight, regulated, fine-tuned coordination of pathways and processes, including the fundamental role of the FGF axis and its crosstalk with other signaling cascades.

Finally, FGF signaling mediates angiogenesis and osteogenesis, two closely related processes of bone formation (Shahi *et al.* 2017). Hence, the relevance of its therapeutic application in cancers involving bone.

## Cancer progression to established bone metastases

Following local progression, cancer cells may acquire traits that allow them to escape the local site and disseminate via the blood stream (circulating tumor cells (CTCs)). Once they reach a distant site (disseminated tumor cells (DTCs)), they may get mechanically trapped in the capillary beds (passive arrest) or may specifically stay in certain organs by, for example, receptor-mediated tropism (active arrest). It is worth noting that normal bone houses the hematopoietic stem cell (HSC) niche, comprised by hematopoietic and mesenchymal cell populations, which provide homing signals to HSCs and regulate HSC self-renewal (Taichman *et al.* 2010). It has been suggested that DTCs can precondition the metastatic niche and compete with and occupy the HSC niche to facilitate metastasis (Decker *et al.* 2016).

Arrested cancer cells at organ sites (DTCs) may undergo a period of dormancy prior to the development of metastases. The mechanism that makes cancer cells leave the dormant state and start growing is ill-defined and a subject of intense study. It has been proposed that there are signals and factors from the metastatic/HSC niche, including FGF2, that can play a role in exiting dormancy (Decker *et al.* 2016).

As previously mentioned, bone metastases can be osteoblastic, osteolytic, or mixed blastic-lytic. Osteoblastic metastases involve the aberrant formation of new bone by osteoblasts. Among the factors/pathways mediating this process are IGFs, BMPs, FGF, endothelin 1, and WNT ligands secreted by tumor cells. Even though osteoblastic lesions are characterized by the aberrant formation of bone, osteolysis is always present. Therefore, the release of factors embedded in the bone matrix (e.g. IGF, TGFβ) by the bone resorption process will in turn favor the growth of tumor cells. This process is known as the ‘vicious-cycle’ theory (Ell & Kang 2012), which describes a positive feedback loop between cancer cells and bone, as a means of survival and growth in the bone microenvironment.

Osteolytic metastases are characterized by increased bone resorption due to enhanced osteoclast activation. Among the factors implicated in osteoclast activation by cancer cells, either directly or via activation of osteoblasts, are RANKL, PTHrP, and IL-6. Here, as well, a vicious cycle theory explains a positive feedback loop between cancer and bone, in which cancer cells induce the release of factors from the bone matrix that promote tumor cell proliferation and survival (Ell & Kang 2012).

## FGF axis in bone metastases

Alterations in the FGF/FGFR axis found in cancer result either from activating mutations of receptors or from overexpression of ligands or receptors.

### Prostate cancer

Prostate cancer is the second leading cause of cancer-related death in men in the United States (Siegel *et al.* 2017). Patients with advanced metastatic prostate cancer have effective treatment options, but none of them are curative. Androgen deprivation is the most effective therapy, but cancer growth resumes over time in most cases and the disease becomes castration resistant (Watson *et al.* 2015). Bone is the primary site of castration-resistant prostate cancer (CRPC) progression and the main cause of morbidity and mortality of the disease. The underlying mechanisms of progression of metastatic CRPC are diverse and include FGF axis activation (Corn *et al.* 2013). FGF axis abnormalities in prostate cancer have been associated with receptor or ligand overexpression (Wang *et al.* 2019), but mutations of FGF axis components have been detected in only a small fraction of prostate cancers.

**Figure 3:**
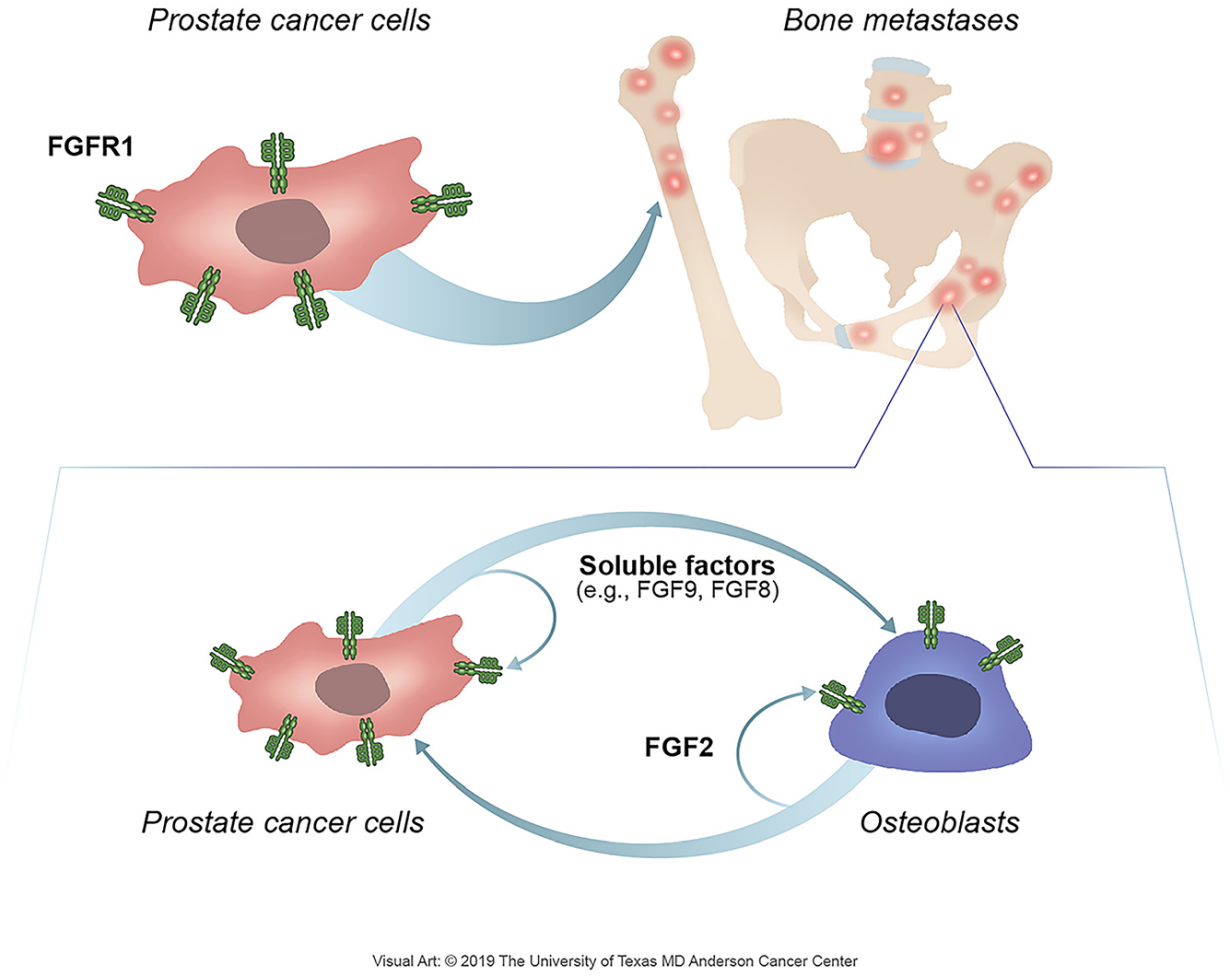
Proposed role of the FGF axis in the progression of prostate cancer cells in bone. FGFR1 expression in prostate cancer cells favors their growth in bone. At the cellular level, soluble factors (e.g. FGF8, FGF9) released by FGFR1-expressing prostate cancer cells mediate an autocrine positive loop as well as paracrine signals to osteoblasts. Tumor-associated osteoblasts express FGFR1 and FGF2, the latter mediating autocrine and paracrine signals, thus constituting a positive feedback loop between prostate cancer cells and osteoblasts. Copyright held by, and used with permission of, The University of Texas MD Anderson Cancer Center.

Overall, 80% of advanced prostate cancers metastasize to bone, where they typically form osteoblastic lesions. However, an osteolytic component is always present (Bubendorf *et al.* 2000). Previous and emerging evidence implicates the FGF signaling axis in prostate cancer bone growth. Given the role of the FGF axis in bone biology, aberrant FGF signaling activation in bone cells would upset the bone homeostasis. It has been reported that expression of FGF8 and FGF9 is significantly increased in human prostate cancer bone metastases compared with the primary site (Valta *et al.* 2006, Li *et al.* 2008). Further, ectopic expression of FGF8 and FGF9 in prostate cancer cells promotes, while blocking FGF9 reduces, the growth of prostate cancer cells in bone (Li *et al.* 2008, Valta *et al.* 2008, Huang *et al.* 2015). These findings implicate FGF8 and FGF9 in the pathogenesis of prostate cancer bone growth. Subsequent studies identified the FGF axis as a candidate target for therapy and suggested that FGF signaling mediates a positive feedback loop between prostate cancer cells and bone cells (Wan *et al.* 2014). It was also shown that blockade of FGFRs with dovitinib (TKI258, Novartis Pharma), a receptor tyrosine kinase inhibitor (TKI) with potent activity against FGFR and vascular endothelial growth factor (VEGFR), has clinical activity in a subset of men with CRPC and bone metastases (Fig. 3) (Wan *et al.* 2014). A recent study confirmed the role of the FGF axis in the pathogenesis of metastatic prostate cancer (Bluemn *et al.* 2017).

A previous report showed that *Fgf2* expression is increased in tumor-associated bone cells in an experimental model (Wan *et al.* 2014). Results of a recent study indicate that high FGF2 levels in osteoblasts (secondary to *Tgfβ* receptor 2 (*Tgfβr2*) loss) promote prostate cancer bone metastases in mice (Meng *et al.* 2018). Prostate stromal cells express biologically relevant levels of FGF2, and therefore the increase in FGF2 in the bone microenvironment may promote prostate cancer cell growth by providing a prostate-like environment (Kwabi-Addo *et al.* 2004, Pecqueux *et al.* 2018). Together these studies suggest that loss of *Tgfβr2* expression in osteoblasts enables FGF2-mediated crosstalk with prostate cancer cells and promotes bone metastasis (Meng *et al.* 2018). In support of these studies, it has been shown that loss of TGFβR2 occurs in the bone marrow of 77% of bone-involved prostate cancer cases examined. Further, knockout of *Tgfβr2* in mouse stromal fibroblasts results in earlier tumor development in intratibial injections in mice (Li *et al.* 2012).

Lastly, a recent study using experimental systems indicates that depletion of FRS2α (a main signal transducer of FGF signaling) in human or mouse prostate cancer cells results in reduced angiogenesis and impaired tumor growth in bone (Liu *et al.* 2016). These results are in alignment with the known role of the FGF axis in angiogenesis attributed to the mitogenic effect on endothelial cells.

Together the evidence discussed suggests that FGF signaling mediates autocrine and paracrine signals between prostate cancer cells and bone cells.

Based on the knowledge outlined, clinical trials with FGFR inhibitors in men with metastatic prostate cancer are ongoing. However, based on prior experience on clinical trials of inhibitors targeting aberrantly activated pathways in CRPC, long-term treatment responses occurred in only a subpopulation of patients and predictors of treatment response have yet to be validated (Zafeiriou *et al.* 2016). Therefore, it might be expected that a monotherapy with an FGFR inhibitor may not accomplish the desired significant control of prostate cancer progression, and it is essential to identify combination therapies that may optimize efficacy.

### Breast cancer

Breast cancer is the most common malignancy among women worldwide. Breast cancer is classified into different molecular subtypes based on expression of hormone receptors (HR): estrogen receptor and/or progesterone receptor and human EGF receptor 2 (HER2), also known as receptor tyrosine-protein kinase erbB-2 (Horton *et al.* 2018). Among the five major subtypes are estrogen receptor-positive and triple-negative (HR-negative and HER2/neu-negative (TNBC)). Bone metastases occur in about 70% of cases and are the most common site of disease recurrence, negatively impacting patient survival, morbidity, and quality of life (Weilbaecher *et al.* 2011). Breast cancer bone metastases generally produce an osteolytic phenotype by secreting factors that activate the bone-resorbing cell, the osteoclast. It has been suggested that FGFs, among other growth factors (e.g. TGFβ, IGF), are released from the bone matrix during bone resorption, which contributes to the vicious cycle process, originally defined in the context of breast cancer bone metastasis (Guise 2002). FGFR1 gene amplification, which occurs mainly in estrogen receptor-positive breast cancer, represents the most frequent genomic aberration of the FGF axis, whereas amplification of *FGFR*s and *FGFR*-activating mutations are uncommon (Perez-Garcia *et al.* 2018). However, the status of these genomic alterations and the role of the FGF axis in breast cancer bone metastases has not been studied.

Experimental studies indicate that the incidence of bone metastases and growth of osteolytic breast cancer cells is impaired in osteoclast-specific *Tgfβr2* knockout mice, and this phenotype is rescued by FGF2. Subsequent correlative analysis of human samples indicate association between the expression of TGFβR2, pSMAD-2, and FGFR1 in breast cancer cells and osteoclasts (Meng *et al.* 2016). Accordingly, it was shown that secreted FGF ligands from breast cancer cells can promote differentiation of osteoclasts, that breast cancer cells enhance osteoclast function in an FGFR-dependent manner, and that this effect is reduced when FGFR is inhibited (Aukes *et al.* 2017). It is worth noting that the experimental studies outlined have been done mainly using TNBC cancer models. It remains to be seen if this holds true when using models of other breast cancer subtypes such as estrogen receptor-positive.

### Lung cancer

Of the two main types of lung cancer, small cell lung cancer (SCLC) and non-small cell lung cancer (NSCLC), which accounts for 85% of lung carcinomas, *FGFR1* is amplified in 22% of squamous cell lung carcinomas, a subtype of NSCLC (Katoh & Nakagama 2014). Further, preclinical studies have shown FGFR-altered NSCLC cell lines respond positively to FGFR inhibitors (Hashemi-Sadraei & Hanna 2017). Lung cancer bone metastases, which occur in 30% to 40% of cases, are typically osteolytic, and the ‘vicious cycle’ defined for other malignancies has also been implicated in this disease (D’Antonio *et al.* 2014). The release of growth factors from the bone matrix in this context includes FGF ligands.

### Multiple myeloma

Seventy percent of patients with multiple myeloma present with bone metastases at diagnosis and 90% will progress and develop bone lesions that are typically osteolytic (Bataille *et al.* 1989).

Fifteen percent of multiple myeloma patients present with a t(4:14) translocation that results in overexpression of FGFR3. Therefore, clinical trials targeting the FGF pathway have been under study. These included the receptor TKI, dovitinib (TKI258), which showed signs of increased progression-free survival and disease stabilization, but exhibited severe adverse effects on patients (Porta *et al.* 2017). Other agents that could minimize these off-target effects by being more selective, including monoclonal antibodies (i.e. FGFR3-specific antibody MGFR1877S, hampering receptor dimerization) and more specific inhibitors (i.e. pan-FGFR inhibitor JNJ-42756493 (Janssen pharmaceuticals) and NVP-BGJ398 (Novartis)), are currently under evaluation (Porta *et al.* 2017). How these agents have direct beneficial effects in bone metastases in particular have not been described in detail, and the bone-specific research area in this context remains relatively unexplored. Thus far, one laboratory-based study has shown that the FGFR1 inhibitor NVP-BGJ398 blocked cell growth and blocked the induction of RANKL in co-culture studies of multiple myeloma cells with neonatal mouse calvarie (Suvannasankha *et al.* 2015).

### Bladder cancer

Urothelial or transitional cell carcinoma is the most common type of bladder cancer, and approximately 30% of its metastases are to the bone (Bellmunt *et al.* 2010). Alterations in FGFRs are frequent in bladder cancer. Primarily, *FGFR3* mutations are found in non-invasive, as well as advanced, metastatic bladder cancer (di Martino *et al.* 2016). Recently FDA approved an FGFR inhibitor to treat locally advanced or metastatic bladder cancer (Alhalabi *et al.* 2019).

High levels of FGF2 have been detected in invasive bladder cancers. Only a correlative study has aimed to explain the molecular mechanism (epithelial to mesenchymal transition, increased proliferation, and trigger of immune checkpoint) for FGF2-mediated poor prognosis (McNiel & Tsichlis 2017). Once again, it is worth noting that no studies have focused yet on the role of FGF specifically in bone metastases in this disease.

## Conclusions

Cancer metastases to bone remain a therapeutic challenge in most cases. Therapy development for bone metastases requires a deep understanding of both bone biology, tumor biology, and the role of this interaction in the pathogenesis of cancer progression. The present review integrates our current knowledge of normal bone biology with that of the FGF axis in bone homeostasis and bone metastasis providing a conceptual framework to develop FGF blockade for cancer metastases in bone. This is particularly relevant given the morbidity and mortality associated with cancer metastasis to bone and the fact that new drugs targeting the FGF axis are now available.

## Declaration of interest

The authors declare that there is no conflict of interest that could be perceived as prejudicing the impartiality of this review.

## Funding

This work was supported in part by the Prostate Cancer Foundation, generous philanthropic contributions to The University of Texas MD Anderson Moon Shot Program, Cancer Center Prostate Cancer SPORE (NIH/NCI P50 CA140388-08), and DOD-PCRP (W81XWH-14-1-0554).
